# Recent Innovations and Developmental Considerations in Mentalization‐Based Therapy for Adolescents: A Case Illustration

**DOI:** 10.1002/jclp.70071

**Published:** 2025-11-26

**Authors:** Madeleine Allman, Carla Sharp

**Affiliations:** ^1^ Department of Psychology University of Houston Houston Texas USA; ^2^ Centre for Development Support University of the Free State Bloemfontein South Africa

**Keywords:** common factors, early identification and prevention, mentalization‐based therapy for adolescents, personality disorder, treatment

## Abstract

Mentalization‐based therapy for adolescents (MBT‐A) is a treatment modality with an evidence base for young people struggling with borderline personality disorder, depression, and self‐harm. Recently, several innovations to MBT‐A have been described to expand its utility to new populations. In this paper, we first describe the basic structure of MBT‐A and its interventions. Second, we describe developmental considerations of working with adolescents. Third, we describe recent innovations to MBT‐A which expand its utility to new populations: (1) nosological reform toward a dimensional model of psychopathology, (2) mentalizing as a transdiagnostic factor for all adolescents receiving psychotherapy regardless of specific disorder or treatment modality, and (3) an explicit focus on the social context of the adolescent outside of the therapy room. We use case material to demonstrate the basic MBT‐A structure and highlight the recent innovations which expand its utility.

## 1 Introduction

Mentalization‐based therapy (MBT) is an evidence‐based treatment designed to enhance an individual's ability to mentalize, defined as the ability to understand oneself and others in terms of mental states such as thoughts, feelings, intentions (Bateman and Fonagy 2016). Originally developed for use with patients with borderline personality disorder (BPD) (Fonagy 1991), MBT's use with adolescent personality challenges was elaborated (Bleiberg 2002), implemented (Sharp et al. 2009), and rigorously evaluated in a randomized control trial (Rossouw and Fonagy 2012). Since then, MBT‐A has demonstrated positive treatment effects in self‐harm behavior, depression, and BPD symptoms across a variety of treatment contexts (for reviews see Hajek Gross et al. 2024; Jørgensen et al. 2021). Currently, MBT‐A's utility is being expanded to new populations and disorders. Our aim in this paper is to first describe the basic structure of original MBT‐A and its key interventions, then highlight developmental considerations specific to adolescents. Then, we describe recent innovations to MBT‐A which expand its potential use with broader populations because of nosological reform and cross‐diagnostic utility (Sharp and Rossouw 2024). Finally, we illustrate both MBT‐A basic principles and innovations with case material to bring to life the value MBT‐A holds for supporting young people and their families.

### 1.1 Mentalization‐Based Therapy for Adolescents (MBT‐A): Basic Structure, Interventions and Developmental Considerations

Following adult MBTs, MBT‐A contains three main phases: Assessment, Initial Phase (mentalization‐based formulation, psychoeducation, crisis planning), the Middle Phase (enhancing mentalizing in adolescent client and family, supporting impulse control), and Final Phase (emphasis is on building independence, stability, and the end of treatment). As with MBT with adults, MBT‐A's goal is to enhance mentalizing capacity in the client during therapy and in their natural environment. To reach this goal, a therapist and client work collaboratively to elaborate the client's thoughts, feelings, reflection on the impact of events, relationships and experiences. Throughout this process, the therapist uses a mentalizing stance of curiosity, uncertainty, and not‐knowing, to enhance the client's ability to reflect on their own and others' minds, and to develop flexibility of mind in managing complex interpersonal situations as well as the impact of a developing sense of self. For detailed descriptions of mentalization‐based approaches, see (Sharp and Bevington 2022; Sharp and Rossouw 2024).

In applying the basic mentalizing interventions to adolescents, several developmental considerations are taking into account. First, adolescence is a sensitive developmental period characterized by increased social demands including a changing and increasingly more complex social environment, specifically the increased importance of peer relationships, and changing relationships with attachment figures/parents (Desatnik et al. 2023). Social cognitive capacities, such as mentalizing, are rapidly developing to keep pace with the increasing demands and challenges of the adolescent environment (Andrews et al. 2021). Despite their rapid social cognitive development, adolescent mentalizing abilities are unstable and are especially prone to breakdown under stress (Fonagy et al. 2002). Adolescents are more likely than adults to slip into “non‐mentalizing modes,” which are ways of thinking that make it hard to reflect on one's own or others' thoughts and feelings (Sharp and Rossouw 2024). For example, in *psychic equivalence*, a young person may believe what they are thinking and/or feeling reflects reality (i.e., “If I feel rejected, the other person must not like me”). In *teleological thinking*, an adolescent believes that only concrete, observable actions, instead of mental states, relate to reality (i.e., “You only care if you stay with me all night”). For a young person in *pretend mode*, the way they express their thoughts and feelings appear disconnected from the content they relay (i.e., discussing strong emotions with in an intellectualized, abstract way rather than connecting with the feeling). These non‐mentalizing modes serve a protective function for adolescents‐ such as using pretend mode to cope with intense shame or self‐consciousness. A therapist working with an adolescent should both interrupt the unhelpful pattern of falling into non‐mentalizing modes and also recognize if there is a protective function for the young person. Above all, the therapist should remain curious and open to the adolescent's subjective experience, resisting any temptation to “know” what the adolescents is thinking or feeling or the motivations behind thoughts, feelings and behaviors.

A second developmental consideration in working with adolescents is that they may struggle with the generalization of mentalizing capacities across environments and may particularly struggle with cognitive constraints of emotion regulation and cognitive flexibility at the same time (Mills et al. 2016). Therefore, it is very important, when working with adolescents, to stay in the “here and now” and deal with present issues of emotional salience to the adolescent. Starting a session with the question as to what might be emotionally salient from the past week's events is a good way of setting the stage for staying in the here‐and‐now with adolescents. Guarding against discussions over generalities and staying close to specifics in working with adolescents prevent falling into pretend mode with adolescents. Adolescents will have less capacity to meaningfully abstract patterns so staying in the here‐and‐now is critical for generating emotionally salient content during session. It is only through the experience of authentic emotion during session that the necessary mentalizing skills to digest that affect can be practiced and developed.

Third, identity formation is a major task of adolescence (Kroger et al. 2010). From early adolescence onward, young people move from an identity based on identifications, through an exploration process, to a new configuration of who they are and how they fit into the world (Erikson 1959). A mentalization‐based therapist must be acutely aware of this “work” and must explicitly support an adolescent client's identity formation and agency in treatment (Sharp 2020; Sharp and Wall 2021; Sharp, Vanwoerden, and Wall 2018). Given that most adolescents still live at home with their parents, treatment should focus on mentalizing within the active and emotionally relevant attachment relationships with their caregivers, peers and intimate partners. The attachment context is highly significant for adolescent identity development and, for clinical adolescents, often fraught with conflict and misunderstanding. It is essential to leverage the family and peer context to practice mentalizing and shift away from coercive or destructive interpersonal cycles. Indeed, the fact that the “adult management” of these relationships are “new territory” for the adolescent can be played up in a tentative way to set up “experiments” to practice adult management of complex situations.

Finally, adolescence is characterized by increased environmental demands including social and societal pressures. While by no means ubiquitous, these changes often contribute to feelings of loneliness and increased vulnerability to mental health difficulties (Von Soest et al. 2020). This may be especially true for adolescents who do not follow a typical developmental path. Loneliness is often obscured and hidden out of shame; thus, a mentalization‐based therapist would be one who slowly and tentatively probe the extent of experienced loneliness and the potential shame associated with that against the background of perceived connectedness of others. Adolescents with personality disorder feel misunderstood and unseen; it is therefore essential for clinicians working with adolescents to instill hope in the potential for change and a positive future. Parents and adolescents are in the unique position to alter the trajectory of the young person to a more positive outcome with early intervention (McGorry and Mei 2018).

### 1.2 Innovations in MBT‐A to Enhance Clinical Utility With Adolescents

Several recent innovations advance the MBT‐A approach and enhance its utility with adolescents by expanding the conceptualization of who may benefit from MBT‐A (Sharp and Rossouw 2024). First, research and debate among experts have pointed toward nosological reform in personality disorder diagnosis, away from diagnostic categories and toward the dimensional Alternative Model of Personality Disorder (AMPD). While some models retain categorical diagnoses, central to the new AMPD and ICD‐11 formulations of personality disorder is the common “core” of all personality pathology—that is, maladaptive self and interpersonal functioning. Elsewhere, we have argued that it is in particular the developing sense of self that drives personality (and interpersonal functioning). It just so happens that the consolidation of self and identity is the major developmental task of adolescence. This places self development at the center of personality functioning—an argument for which there is increasing empirical support (e.g., Sharp et al. 2025). This view on personality and personality development shifts the treatment focus away from traits, and following McAdams' (2015) model for personality development, describes the evolution from early temperamental traits to personality traits, then in middle childhood to goals and values, then in adolescence to develop a “sense of self” through reflection (e.g., Sharp, Kerr, and Channen 2021; Sharp, Bo, and Chanen 2025). In adolescence, because individuals for the first time begin to reflect and “make sense of” their traits, goals, and values, with sophistication afforded by increased metacognitive capacity (multicomponent ability to understand and reflect on mental states; Semerari et al. 2014), they are able to integrate and “bind” their personality in a coherent whole. We have argued that for this binding to occur, mentalization is the critical “glue” (Sharp and Wall 2021; Sharp 2020). If this is so, the move towards a dimensional model that places maladaptive self and interpersonal functioning (i.e. personality functioning) point to mentalizing's essential mechanistic role in achieving healthy personality functioning. This conceptualization of personality development is also useful clinically, as families are more able to understand the core of personality challenges (difficulty in understanding self and others), rather than isolated symptoms (emotion dysregulation, suicidality).

A second advancement in the MBT‐A approach is the realization of mentalizing as a cross‐diagnostic construct and enhancement of mentalizing as a common factor in all effective adolescent psychotherapy (Lüdemann et al. 2021; Sharp and Bevington 2022), and can also be situated within the broader psychotherapy integration literature. Recent work on common factors has emphasized that effective treatments share relational processes such as alliance, empathy, and collaborative meaning‐making, which account for a significant portion of therapeutic change across modalities (Flückiger et al. 2018; Norcross et al. 2019). In this respect, MBT‐A's emphasis on mentalizing may be understood as an integration‐friendly stance that operationalizes these common factors through a focus on reflective functioning and epistemic trust. Rather than positioning itself as a rival to other evidence‐based interventions, MBT‐A extends the common factors framework by providing a developmental and mechanistic account of how therapeutic attunement translates into increased capacity for social learning and healthier adaptation in the adolescent's broader ecology. Furthermore, mentalizing has been described as the central psychological mechanism through which personality functioning is achieved, and disruptions in mentalizing contribute to a wide range of psychopathology. When adolescents are able to mentalize effectively, they can maintain a coherent sense of self and engage in authentic, mutually satisfactory relationships with others. For instance, consider an adolescent who feels disappointed after receiving a lower grade than expected on a group project. Rather than concluding that the teacher is unfair or that peers are angry with them, the adolescent reflects: *“I feel upset and I think it may be because I wanted to do better. I'm going to try and figure out what the teacher is trying to convey and see what my classmates think about our grade.”* This reflective stance allows the adolescent to regulate their own emotions, preserve a stable sense of self‐worth, and approach peers in ways that sustain trust and connection. Such examples illustrate how enhanced mentalizing capacity directly supports both self‐functioning and interpersonal functioning, underscoring its importance as a developmental target for all adolescents. These effects of enhanced mentalizing are beneficial for adolescents, regardless of whether they meet diagnostic criteria for personality disorder or another disorder. Personality functioning in the AMPD describes a spectrum of personality functioning which ranges from healthy to severe dysfunction. As mentalizing is the central process by which healthy personality functioning is developed, it is essential to use mentalization‐based approaches across the spectrum of severity, even to address subclinical thresholds of personality dysfunction, and across a variety of disorders, which may contribute to functional impairment. This aligns with calls for precision mental health approaches that focus less on “one‐size‐fits‐all” protocols and more on tailoring treatment to individual trajectories, needs, and contexts (Nye et al. 2023). By working at the level of personality functioning (e.g., DSM‐5 AMPD Criterion A) and explicitly scaffolding the adolescent's capacity to engage with their social environment, MBT‐A offers a flexible framework that can be adapted across diagnoses while still providing a mechanistic rationale for intervention. In this sense, MBT‐A anticipates and contributes to the emerging movement toward adaptive, mechanism‐focused interventions that prioritize personalization of care over rigid adherence to disorder‐specific protocols (Taubner and Sharp 2024).

A third innovation in MBT‐A the notion of enhancing mentalizing outside of the therapy room and outside of the client‐therapist relationship. Luyten and colleagues have expanded mentalization‐based theory to include communication systems beyond the client‐therapist relationship (Luyten et al. 2020). Indeed, Luyten emphasizes that being understood within the client‐therapist relationship, and developing *epistemic trust* (i.e., an individual's willingness to consider information from someone as trustworthy, generalizable, and relevant to the self; Fonagy and Campbell 2017), are foundational to learning in the social environment. This consideration is supported by other findings that adolescents with increased trust in others (peers, parents) in addition to enhanced mentalizing abilities, show improved clinical outcomes (Bo et al. 2017). The mentalization‐based therapist is only the facilitator that “opens the door” to learning and the natural salutogenic effects of healthy relationships for the adolescent in their natural environment. By practicing the serve‐and‐return in therapy, the resources in their social environment can then be more readily available to the adolescent. In other words, epistemic trust allows individuals to move beyond rigid defensiveness and to re‐engage with the social world as a source of reliable information. Being understood in the therapeutic relationship can establish epistemic trust, enabling adolescents to benefit from learning opportunities and corrective experiences outside of therapy. For example, a young person with low epistemic trust may struggle to perceive adults' input as reliable or relevant, leaving them more vulnerable to risky peer influences with substance use or skipping school to gain acceptance. Through treatment, this young person's epistemic trust could be gradually restored via repeated feelings of being understood and recognized by the therapist. The adolescent is able to understand the therapist's feedback as relevant, supportive, and generalizable to their life choices, and reconsider whether other adults in their life could be trusted.

In what follows, we use case material from a naturalistic clinical case from our clinic to demonstrate adherent MBT‐A keeping in mind both the basic MBT‐A interventions, as well as the recent innovations described here. While we routinely make use of measures in our clinic to assess baseline, process and outcome, this case was transferred from another clinic where measures are not routinely used.

## 2 Case Illustration

### 2.1 Presenting Problem & Client Description

Jamie is a 14‐year‐old African American girl referred for outpatient therapy in a personality disorder specialty clinic. She was previously hospitalized following a suicide attempt, she then completed 2 months of residential treatment, and a 3‐month intensive outpatient program focused on building emotion regulation skills. Upon referral, Jamie was struggling with emotional “outbursts,” frequent arguments with her parents, substance use, and self‐injurious behaviors including cutting and burning.

Jamie was adopted into a high SES White family when she was 2‐years of age. She reported a history of severe neglect and abuse in her biological family, saying that she was “taken” from her parents when she was a baby. Jamie and her parents talked openly about the adverse conditions she lived in with her biological family. Jamie described that she “hated” her biological parents and that they “didn't deserve” to have children. She had no contact with her biological family following her adoption. In early childhood, Jamie was reportedly delayed in several developmental milestones such as walking independently and using language to communicate. She received speech and physical therapy and “caught up” with her developmental milestones. In fact, Jamie excels in academics and has maintained high grades throughout her emotional difficulties and mental health treatment.

Jamie and her mother described longstanding difficulties for Jamie in mood and in social interactions. At age 9, she began receiving outpatient therapy for depressive symptoms including social isolation, intense irritability, and low mood. Jamie worked with several therapists. Her parents reported that they often struggled to see any positive benefits for Jamie from therapy, so would discontinue treatment after a few months and start over with another specialist in hopes of an improved outcome. Jamie discussed often struggling to know who she “really” was and felt that she was “always” changing herself based on her friends at school. Jamie reported significant struggles in connecting authentically to her own emotions or emotions of other people. In disagreements with friends or with her parents, Jamie frequently felt as if she had “no idea” what was happening in others' minds, or that other people were “always overreacting.” With peers, Jamie described that she often felt left out or like an “outsider” due to her race and her experience as a “Black girl in a White family.” Jamie described feeling misunderstood by her parents frequently. Intense arguments occurred, especially between Jamie and her mother when Jamie “acted out” by breaking rules or when Jamie's mother thought Jamie was lying.

At age 13, Jamie attempted suicide by taking a handful of prescription medication she found in her family's medicine cabinet. At the time, Jamie was struggling with her group of friends at school “dropping her” after she had an argument with a friend. Jamie stated that she tried to talk to her parents about how lonely she was feeling, but felt she was met with “a lack of understanding.” Her parents took away her phone so that she would calm down. Instead, Jamie felt more isolated from her peer relationships and even more desperate. She was hospitalized by her parents and referred to residential treatment. During her residential treatment, Jamie was diagnosed by a psychiatrist with borderline personality disorder (BPD), before coming to our clinic. This is quite common despite the growing evidence in support of the developmental relevance of the AMPD. However, given our own clinic's focus on dimensional diagnosis, the therapist built into the psychoeducation phase education around the value of dimensional approaches for in early intervention. Jamie reported that she did not identify with the BPD diagnosis, because she was not “really suicidal,” she just didn't have any other ways to show her parents how much pain she was in. Also, she noted that she just “hated” the diagnosing clinician, and that she “showed her worst side on purpose” during their meetings.

Jamie described some positive impacts of previous therapeutic experiences. She felt connected to several of her previous therapists, stating that they actually “got” her perspective. Both Jamie and her mother noted that skills‐based approaches had given them tools to use in the moment when Jamie was struggling with her emotions. However, they both also described that none of her previous treatment had helped them with the “underlying” issues for Jamie and her family. The family felt desperate for help as the felt like the window for placing Jamie on a different trajectory was closing.

### 2.2 The Therapist

The therapist working with Jamie and her family was an upper‐level Clinical Psychology graduate student. She is a White woman. She had previous experience in mentalization‐based approaches via formal MBT‐A workshop training and received ongoing supervision by a certified MBT‐A clinical supervisor. She is highly interested in working with children and families, especially in the early identification and treatment of personality problems. She is supervised by licensed clinician who is an expert in mentalization‐based modalities.

### 2.3 General Clinical Formulation

Jamie's difficulties are best understood as her attempts to manage negative emotions by avoiding them completely in the moment (using self‐injurious behaviors such as cutting or burning to focus on the physical sensation, or using drugs or alcohol, rather than the emotional experience). Also, in attempts to manage intense emptiness and loneliness, Jamie acts out with impulsive, risky, or hostile behaviors. These include engaging in risky sexual encounters to “feel close” to others, skipping class to use drugs and alcohol with friends to facilitate social interaction, or bullying peers at school to “get in” with other groups. While Jamie feels temporary benefits from these risky behaviors (feeling connected to friends at school, being perceived as “cool” for getting a tattoo), she struggles with the adverse consequences. Jamie was often in trouble with her parents, getting grounded or her phone taken away as punishment for skipping class or getting caught with alcohol. Jamie attempted to hide her risky behaviors from her parents to avoid getting in trouble. This resulted in Jamie feeling “on her own” to manage any other consequences of these actions such as regretting a tattoo and attempting to get healthcare after contracting a sexually transmitted infection. Her feelings of being an outsider are amplified because she was adopted and identifies as African American, while her parents are White. Throughout her childhood, Jamie worried that her parents would “just return her” to the adoption agency when they were angry with her.

In treatment, Jamie's parents identified the goal of her gaining “life skills” to be able to manage her emotions. They hoped she would not engage in any more suicidal or self‐harming behaviors, and they hoped that she would have a more “normal” social life as a teenager with less intense peer relationships and risky behaviors. Jamie wanted to learn more about who she was and how to choose better relationships, so she wouldn't get hurt so often.

MBT‐A was deemed appropriate for Jamie given her age and presenting problems with suicidality, self‐harm behavior, interpersonal difficulties, and depressed mood. Her intense emotional reactions and behavior to “escape” from her feelings suggested underlying problems in mentalizing herself and others. Additionally, MBT‐A's developmental focus on Jamie's attachment context with her parents was a good fit because of the struggle for Jamie and her parents to understand each other and communicate openly.

### 2.4 Course of Treatment

Jamie's treatment followed the standard structure of MBT‐A (Table 1) with weekly individual sessions with Jamie and monthly (every 4th week) sessions together with Jamie and her parents over 12 months.

**TABLE 1:** Structure of MBT‐A.

Assessment Initial phase ○ Formulation, contract and crisis plan with involvement of parents ○ Psychoeducation, including both adolescents and parents Middle phase ○ Improving mentalizing and impulse control ○ Enhance awareness of mental states of others ○ Help with adolescent tasks and milestones ○ Improving family mentalizing Final phase ○ Increase independence and responsibility ○ Consolidate stability ○ Develop follow‐up plans ○ Understanding and processing of the meaning of the ending and focus on affective states associated with loss ○ Discharge and liaison with partner organizations

#### 2.4.1 Assessment

In MBT‐A, assessment of mentalizing capacities is an intrinsic part of the treatment model and continued throughout the intervention. The first few sessions were focused assessing Jamie's mentalizing capacities to get a sense of what is typical for Jamie in terms of mentalizing, how she is able to understand and attune to others, how she understands herself, where she may struggle with understanding herself or others, and the circumstances under which these struggles arise.

#### 2.4.2 Mentalization‐Based Formulation

The assessment of mentalizing is followed by a mentalization‐based formulation that formulates the case in mentalization‐based terms, and a formulation letter that is shared with the adolescent. This formulation is quite unique in that it directly communicate what is noticed in terms of mentalizing capacity, as well as the impact the adolescent has on the therapist.

#### 2.4.3 Formulation

Despite Jamie's interest and apparent willingness to engage in therapy, she frequently struggled to describe her internal experience using mental state language. Jamie often described her own emotional state as “fine” or by shrugging her shoulders (pretend mode) when asked how she was feeling. Jamie also demonstrated a tendency to rely on external or behavioral cues from others (telelogical mode), and she struggled to use curiosity or uncertainty about their intentions or minds often assuming that what is in her mind is true for everyone (psychic equivalence). For example, when Jamie's mother became tearful while describing Jamie's previous suicide attempt, Jamie explained that she was “certain” that her mom “cried on purpose” to gain the attention of the session. Other times when asked about why a friend or even the therapist would behave in a certain manner, Jamie struggled to attribute mental states to others' behavior. Jamie similarly struggled to mentalize herself when discussing previous risky behaviors (such as using drugs or having casual sex), stating that she had “no idea” what she was thinking or feeling beforehand. Jamie sometimes exhibited pretend mode when talking about past situations she “was not proud of” and described her strong identity as a religious and values‐based person while her affect was disconnected from the content of her speech, and also did not reflect the fact that she was engaging in behaviors inconstant with her religious beliefs. Overall, Jamie struggled to make sense of herself and others in terms of mental states. She showed a tendency to oscillate between hypomentalizing (overly simplistic explanations about internal states—“I don't know why I got high”) and hypermentalizing instances (over‐attribution of mental states to others—“my mom cried to get attention”).

#### 2.4.4 Initial Phase

During the Initial phase of treatment, Jamie and the therapist worked together on a written letter from the therapist's perspective about what she had learned about Jamie so far in the assessment phase. In plain language, the therapist attempted to show curiosity, authenticity, and non‐authoritativeness in describing what Jamie had brought to therapy. After discussing it with Jamie, the formulation letter was also shared with Jamie's parents. Given the importance of the attachment context for adolescents, it was essential for Jamie's parents to be engaged in her treatment. A crisis plan was also developed together with Jamie and her parents if she felt overwhelmed by her emotions or was feeling suicidal.

### 2.5 Formulation Letter

*Dear Jamie*,

*We have met together for three sessions now and I have learned a lot about you so far. I want to share with you what I feel like I've learned about you and I want you to tell me if it fits with how you see things. We are just getting to know each other and I hope you'll tell me if I don't get things quite right – okay?*

*During our first meeting with your mom, I was struck by how much has happened in your life even though you are only 14 years old. You shared with me about how you were adopted as a toddler into your family and that your biological mom and dad had a lot of problems going on when you were born. You haven't been in touch with your biological parents since you were adopted and you don't even know if they are alive anymore. You shared with me that you feel very angry with them for not taking better care of you and that they were not good parents to you. You told me that you feel you have nothing in common with them and you want to know why they had you if they weren't able to take care of you. I got the impression that thinking about this made you sad and angry, and I wondered if this may be because it feels like it will “never” be resolved. I think you are very brave for talking about this with me*.

*You also told me about how it's been hard to figure out how to fit in with your family, even though you love them a lot. You shared with me that you identify as Black, but your family is White. You told me that this made it hard for you growing up to understand yourself and to know who you really are. You told me that you are “like a chameleon” and can change yourself depending on who you are with at the moment, including at school and with your friends. You told me about how confusing it can be, to act one way in one situation and a totally different way in another. You said you sometimes feel completely alone, even when you are with other people because they don't know the “real” you. This must be very isolating for you and I feel sad thinking about you as being this isolated and alone*.

*You've also described that your emotions change very quickly. You can go from feeling “nothing at all” to feeling extreme anger or sadness. You often struggle to understand what happened to make your mood change so drastically. You say you feel “out of control.” Sometimes to manage these intense emotions like anger or sadness, you use self‐harm like cutting or burning to make yourself “not feel” the emotional pain. Then afterward, you feel shame about self‐harming. If your parents find out that you self‐harm, you usually get in trouble, which you say makes you feel more shame and more alone. Sometimes, in an attempt to cope with this, you have used harsh words with your parents or other people so that they leave you alone, even though you say you feel very lonely and sad at the same time*.

*With your friends at school, you often feel like an outsider because you are Black and most of the kids at your school are White. One way you told me you make friends with other kids is by doing things that sometimes get you into trouble. You say you definitely don't like the consequences of getting into trouble, but at the same time you say it can feel good when you feel accepted and “in it together” with your peers. I can see how you want to feel connected to other people and share experiences with your friends*.

*When I've asked you about what you would like to get out of therapy, you've said that you want to understand yourself better, and that you want to be better at choosing relationships. You feel like you often pick friends and boyfriends that get you into trouble, even when that isn't what you want. Your parents told us that what they want from you in therapy is for you to gain “life skills” to deal with your emotions and work through them without self‐harming or becoming suicidal. The impression I've gotten from meeting you a few times is that you are an extremely bright young person who sometimes feels confused and isolated because of your strong emotions. When you feel lonely or misunderstood, you tend to act quickly, using self‐harm or risky decisions with friends, to help you get rid of those negative emotions. Does this sound right? The kind of therapy I do, called mentalization‐based therapy, helps people to slow down in the moment and look at their thoughts and feelings to try and better manage them. This kind of therapy also helps us think about how we impact our relationships, and how our relationships can impact us. I think this kind of therapy could be helpful to you. What do you think? Would you like to work together in this way?*

*If you agree to continue work with me, I want you to know that I see you as the expert on your life and what will work for you. I see my job as walking alongside you and trying to help us see the situation from different perspectives to see if we can understand it better. I will know if I'm doing a good job if you feel like I am beginning to see things from your perspective. I hope you will tell me when I get it wrong too, so we can learn together. I am really excited to keep working with you*.

#### 2.5.1 Psychoeducation and Dimensional PD Framing

In the Initial phase of treatment, time was also dedicated to psychoeducation, grounded in a dimensional understanding of personality functioning. The therapist discussed, using examples from their sessions, how Jamie demonstrated her ability to mentalize. She also discussed that the capacity to mentalize comes and goes, often depending on stress. Examples were generated for when Jamie felt an intense emotion and “lost it” to understand better how Jamie's mentalizing becomes impaired during “hot” (triggering) situations. Consistent with guidance on psychoeducation in MBT (Sharp and Bevington 2022), the therapist used examples from the “here and now” (that had high emotional salience) and frequently checked in with Jamie to see how she wanted to learn and discuss new concepts and terms in therapy. For example, when identifying times when Jamie was not mentalizing, the therapist asked Jamie how they should refer to that feeling‐ whether by a shorthand term or drawing a face on the whiteboard. Jamie identified that calling it “when [she] lost it” was helpful to her to remember the concept. Other clients may prefer to make notes, draw an artistic representation on the whiteboard, or use other metaphors or methods of learning. The therapist also discussed that Jamie may find it difficult to discuss her emotions, because “sometimes it is easier to just avoid them” (*pretend mode)*.

Additionally, the therapist and Jamie discussed her previous psychiatric diagnoses and how Jamie related to each of the diagnoses. Jamie described that her feeling of being an outsider and struggling with low mood began very early for her in life. When she was 9, she was treated for depressive symptoms including low mood, irritability, and social isolation. She identified with the diagnosis of “depression” and felt that this captured nearly all of her mood symptoms, including her suicidality and self‐harm. Jamie strongly did not agree with the label of BPD for her symptoms. She had met other teenage girls who had “BPD for real” in her previous treatment programs, and did not feel that she fit the diagnosis because she was “aware” that she was changing all the time to fit different contexts. Jamie also asserted that her problems with knowing who she was “made sense” with her racial/ethnic identity in the context of her White family. Finally, Jamie was “absolutely certain” that the psychiatrist from her previous treatment program who diagnosed her with BPD was just “out to get [her]” and wanted to give her a “bad diagnosis” so no one would want to try to help her. The therapist listened with curiosity and used empathic validation‐ especially in understanding the dynamic with her previous BPD diagnosis.

*Consistent with the MBT‐A innovation of nosological reform*, the therapist introduced the DSM‐5 Section III (Level of Personality Functioning; Criterion A) formulation of personality disorder. Together, the therapist and Jamie discussed Jamie's symptoms in developmental terms, emphasizing impairments in self (identity, self‐direction) and interpersonal functioning (empathy, intimacy). “*As a teenager, you're busy trying to put the pieces together of who you are as a person. Some of the things that have happened to you, like being adopted into a White family as someone who looks different than your parents and not making friends easily, have made it more difficult for you to do the work of building the person that you are*.” By discussing her experience in terms of self and interpersonal functioning, Jamie was able to see more of her symptoms explained by personality functioning. By emphasizing the developmental aspect of Jamie's symptoms, the malleability of personality functioning was also emphasized. Because the way Jamie understands herself and other people is currently growing and changing during her adolescent years, it is an ideal time for treatment to address these issues.

The therapist frequently checked in with Jamie and her parents during psychoeducation to ensure understanding and invite dialog. While Jamie and her parents maintained some hesitancy around the personality disorder “label” and stigma associated with the disorder, they agreed that the diagnosis “rang true.” Through this discussion with the therapist, Jamie and her family also discussed that they felt there was hope for her symptoms. Previously, they were told that personality disorder was a “lifelong diagnosis” and that Jamie could never get better if she had personality disorder. This myth was dispelled by sharing some of the recent research on a dimensional conceptualization of personality disorder in youth with Jamie and her family. Overall, psychoeducation focused on a developmental understanding of Jamie's difficulties, rather than a categorical or “fixed” diagnosis. Jamie's parents were also able to understand Jamie's behaviors as manifestations of difficulties in self and interpersonal functioning (i.e., making risky choices to fit in and gain sense of identity with peers‐ rather than willfully disobeying parents). An explanation of Level of Personality Functioning as a new approach to understanding personality functioning, rather than “fixed traits” was provided. This conveys a hopeful message to the family, because it conveys that “change is possible.”

### 2.6 Middle Phase

**FIGURE 1:**
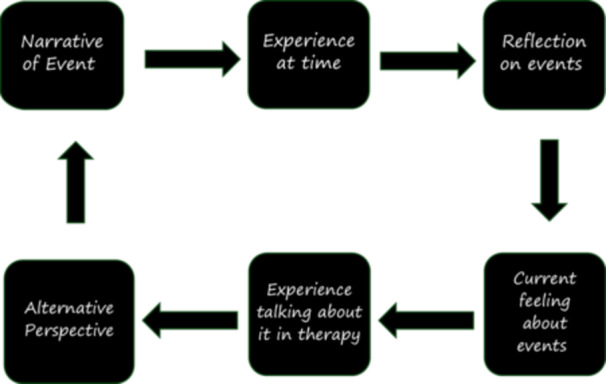
The mentalizing process to “stretch” out the serve‐and‐return which enhances reflective capacity. Adapted from Sharp and Bevington (2022).

In the Middle Phase of treatment, Jamie, her parents, and the therapist worked together to facilitate mentalizing during treatment sessions and outside of the therapy room, using goals collaboratively agreed upon between Jamie and the therapist, (1) Understanding her own emotions better, (2) Choosing better relationships that have less conflict, and (3) Reducing her self‐harm behavior. Using MBT‐A interventions such as empathic validation, affect elaboration, contrary moves, and mentalizing the relationship, the therapist worked together with Jamie and her parents to increase her ability to manage her emotional arousal while mentalizing. The mentalizing loop (Figure 1) is a major tool in devising mentalization‐based interventions. The loop provides a framework to “slow down” and stretch a narrative to promote mentalizing. The loop can be started from any point and followed. An example interchange illustrating the mentalizing loop is below from Middle Phase session after Jamie had been caught skipping school and had been grounded by her parents.

*Jamie: My life is ruined (psychic equivalence). They took my phone and said I can't have it back for 2 weeks. I wasn't even off campus*.

*Therapist: Oh no. I can tell something has really upset you. [empathic validation] What happened? Your parents took your phone? You said you left campus? (clarification)*

*J: No! I didn't even leave campus I was just skipping class in the parking lot. And then they completely overreacted and now my life is ruined (psychic equivalence)!*

*T: Wow, this sounds really infuriating (empathic validation). Hang on though, I'm not following (stop and pause). It's going too fast for me to catch the details of what happened. I want to have a clear picture – can we rewind a little so I can get a sense of how things happened? [request for clarification ‐ rewinding]*

*J: Well, this group of friends were planning on going off campus for lunch. They never invite me but today they did. We were in the car and about to leave the parking lot. We're not technically allowed but we rarely get caught at lunch time. Of course the day I get invited to go with them, a teacher sees us and calls my mom. Then she completely overreacted and now I'm grounded!*

*T: I see, you were going out with some friends you were excited to be included with, but leaving campus is not something you usually do. Then you were seen by a teacher, and the teacher called your mom. Help me again to understand, how did you hear from your mom? What happened next? [clarification]*

*J: We turned the car around and I just went back into school. Our lunch off campus was ruined. Then I went about the rest of my day as usual but I could feel that my mom was mad. When I got home from school, she was waiting for me. She was so angry. She told me that my teacher had called her and that I had to give her my phone and that I couldn't go out for 2 whole weeks! Isn't that insane? She does not want me to be happy or have a social life. She hates me (psychic equivalence). And I hate her*.

*T: I think I'm beginning to understand. Thanks for taking me through it so slowly. So let me see if I got this right. So this group of friends invited you to go off campus which is against the rules but exciting because they were including you. Then you guys got caught by the teacher and your mom found out. And now you're grounded AND you didn't even get to go to lunch. Man, that is a lot to handle in 1 day (empathic validation). How did you manage all that? I can imagine you feeling a lot of feelings at once? … [affect elaboration]*

*J: (tears filling up) I just… wanted to be with my friends. They finally invited me. Then it got ruined… and I can't even talk to them online now because she took my phone… I feel so alone and like I'll never have real friends (mentalizing)*.

*T: Oh Jamie, I'm so sorry – this is a lot to handle (empathic validation). Can you tell me a little more about this thought that you'll never have real friends; that you are feeling very alone?*

*J: Well, I won't be able to talk to them for 2 weeks. That's forever. (psychic equivalence)*

*T: Yes, I can see how you are thinking about it (empathic validation). Can we try something for a bit though? What do you think might happen in these 2 weeks that you have no contact with them?*

*T: I'm not sure… (uncertainty – index of mentalizing coming back online)*.

*J: I don't know for sure either. But I'm wondering if there is a possibility that they may still be your friends (contrary move)… Can we rewind again to when they invited you out to lunch? How did that come about?*

**FIGURE 2:**
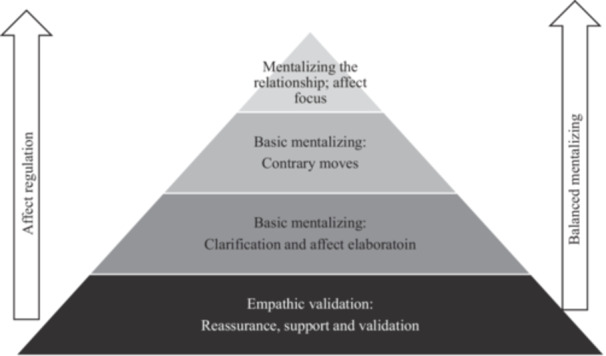
The affect pyramid depicting a spectrum of mentalizing intervention associated with the client′s capacity to regulate affect and to mentalize. *Note:* This diagram organizes the hierarchy of MBT‐A interventions from bottom (empathic validation) to top (mentalizing). Mentalizing capacity is inversely related to emotional arousal. Therefore, therapists must attend to clients′ affect regulation before proceeding with interventions. Clinicians begin with empathic validation to restore affective regulation and then move upward toward clarification, gentle perspective‐challenging, and ultimately mentalizing the therapeutic relationship itself. Movement up or down the pyramid reflects the therapist′s moment‐to‐moment decision‐making based on the adolescent′s current capacity to regulate affect and sustain mentalizing under emotional arousal. Adapted from Sharp and Bevington (2022).

This example shows the increasing level of affect focus and elaboration and introducing mentalizing throughout a session while taking into account the client's emotional arousal levels (see Figure 2).

#### 2.6.1 Using MBT‐A Interventions

Starting with empathic validation, Jamie is able to calm down and reflect on her emotions (lowest level of pyramid). Then the therapist starts to get more details around the narrative, focusing on trying to understand the basic facts around the situation and elaborate Jamie's affect at the time the situation occurred, as well as her current feelings now discussing it in therapy (second level of pyramid). Then the therapist then uses a “contrary move” or intervention to move Jamie out of a prementalizing mode (psychic equivalence: I think I will never have friends, therefore it is true) (third level of pyramid). Mentalizing capacity is vulnerable to collapse with high emotional or physiological arousal. Over time in therapy, Jamie builds skills of being able to “hold” or manage her emotional arousal while still engaging in collaborative, reflective meaning‐making with the therapist. The therapist uses increasingly complex interventions in a sequence to stimulate mentalizing. The highest and most complex intervention includes mentalizing the present relationship or bringing awareness to the impact the client and therapist have on each other (highest level of pyramid).

Jamie's behavior of skipping class and desire to be accepted by her peers at school are not unique symptoms to her personality disorder diagnosis. This treatment focus is highly aligned with the Middle Phase of MBT‐A's stated goal of improving mentalizing and impulse control. Additionally, this interchange demonstrates the utility of MBT‐A beyond only adolescents with personality disorders. Adolescence is a developmental period characterized by normative increases in impulsivity and a social reorientation to valuing peer relationships. As such, all adolescents may benefit from mentalization‐based interventions to help them make sense of their experiences and form their identities. Jamie's enhanced mentalizing capacity helped her to reflect on and make sense of her traits to “bind” her personality into a coherent self. Over time and through many repetitions, Jamie was able to gain skills in slowing down, mentalizing herself and the situation, she became less prone to risky or impulsive decisions. She also gained capacity to connect more effectively with her peers. By slowing down and not overly adapting herself to new situations, Jamie was able to find peers she could more authentically relate to and develop relationships with. *Consistent with the MBT‐A innovation of enhanced mentalizing in the adolescent's natural environment*, the therapist attempted to open the door to epistemic learning in therapy, so that Jamie can continue to benefit from the positive benefits of learning from her social context. The therapist scaffolded how Jamie could generalize the trust they had built in their relationship to other social interactions in her environment (peers, teachers, family members). For example, the therapist helped Jamie reflect on how to approach a peer after conflict or to abstain from risky behavior to avoid getting into trouble. The therapist invited Jamie to reflect on how she might advocate for herself in school settings. These interventions enhanced opportunities for Jamie's epistemic trust to emerge outside of the therapy room and for Jamie to benefit from the natural benefits of her social environment. In contrast to “classic” MBT‐A, which typically focused on Jamie's individual capacity to mentalize in the therapy room, the next‐generation of MBT‐A invites a broader, more salutogenic framing, emphasizing the generalization of mentalizing skills beyond the therapy room. For instance, with peers, Jamie's development of epistemic trust in the therapist was generalized to her peer environment by inviting Jamie to reflect on the impact of some peers versus other peers. These reflections led to Jamie choosing peers that were more reliable sources of social learning. Rather than focusing only on emotion regulation “in the moment,” the therapist explicitly worked with Jamie to reflect on which peers make her feel genuinely understood and safe, and which peers were associated with more risky or destabilizing outcomes. In this way, MBT‐A's emphasis utilizes Jamie's natural social environment not only as a source of information, but also as and vehicle for change.

#### 2.6.2 Supervision

Ongoing supervision is an essential component of MBT‐A, including in the Middle Phase when therapeutic ruptures are common (Fonagy et al. 2023). Given that mentalizing is most vulnerable under conditions of heightened emotional arousal, the therapist struggled at times when she “got stuck” or lapsed into a non‐mentalizing mode with Jamie. These occurrences happened most frequently when Jamie would “lash out” and become angry with the therapist. On one occasion when discussing Jamie's skipping school, she told the therapist “You don't get it! You're just like my parents—always telling me I'm doing something wrong!” (psychic equivalence‐ equating the therapist's intervention with rejection). The therapist noticed the lapse into a non‐mentalizing mode and potential rupture in the therapeutic relationship; she paused and validated Jamie's concern with curiosity “Oh no—I think something happened here. I want to slow down here because my goal is to understand your perspective, not to judge, and I think I may have come across wrong.” In supervision, the therapist discussed her own feelings of whether she had “pushed” Jamie too hard. They discussed that heightened emotional arousal could disrupt both the client and therapist ability to mentalize and that slowing down was a beneficial intervention. However, perhaps taking note of Jamie's non‐verbal cues, noticing her strong affect earlier and employing empathic validation earlier in the session would have helped to keep Jamie's emotions at a level where she continued to mentalize. They also discussed the potential rupture as an opportunity to understand each other better.

##### 2.6.2.1 Final Phase

The Final Phase of MBT‐A is concerned with Jamie's independence, responsibility, stability in her life, and planning to maintain the gains that have been made during treatment. An explicit focus of final treatment sessions includes the ending of therapy and processing the experience of ending the therapeutic relationship. Consistent with recommendations from mentalization‐based therapy scholars, a termination formulation was written together with Jamie. The therapist asked her to reflect on what has been achieved and what needs further work, whether there is anything Jamie needs to address in her relationship with the therapist, how therapeutic progress can be maintained or further developed in the future (Juul et al. 2020), and relapse prevention (Sharp and Bevington 2022). By opening the door for Jamie to authentically engage with the therapist in their relationship, Jamie began exploring authentic relationships in her natural environment with friends, teachers, and her parents. In this way, Jamie will continue to benefit from the salutogenic effects of social resources in her socio‐ecological context.

Jamie noted that one of her most important “tools in her toolbox” from her work in MBT‐A was learning how and when to use emotion regulation skills. In previous treatment programs, Jamie had learned several behavioral skills to help her manage her emotions (e.g., dialectical behavior skills of ice diving or self‐soothing). Yet, she struggled with knowing when to use them or more often “thinking of it too late” after she had self‐harmed or engaged in a different risky behavior. Jamie described that through the mentalization‐based intervention of rewinding a scenario to understand how she was feeling before she engaged in a risky behavior or got into trouble, she was more able to see where her emotion regulation skills would fit. Skills such as deep breathing or progressive muscle relaxation were also utilized during sessions when emotional dysregulation became too high to engage in mentalizing. This practice of incorporating skills from other modalities (such as Dialectical Behavior Therapy or Cognitive Behavior Therapy) is highly useful with mentalization‐based interventions. *Consistent with the MBT‐A innovation stating that mentalizing is a common factor across all psychotherapies*, Jamie's slowing down to understand the function of an emotion regulation skill enhanced its utility and her likelihood of future use.

### 2.7 Outcome and Prognosis

Despite the severity of Jamie's situation at the outset of therapy (suicidality, self‐harm, impulsivity, and difficulty in relationships), her prognosis is positive. She and her parents worked in treatment to change their way of relating to one another to focus more on “saying what they mean and meaning what they say.” In other words, they built a mentalizing culture in their family structure. Jamie's remains a sensitive young person with strong emotions and occasional confusion about the mental states of others. However, she has learned to mentalize herself and “slow down” when she gets confused, rather than acting urgently in the moment. As a result, she has found herself engaged in more developmentally appropriate peer relationships with fewer instances of risky or externalizing behavior. When she feels overwhelmed, she has demonstrated the ability to ask her parents for help. And in turn, her parents have begun to respond with curiosity and warmth, rather than being directive or using harsh consequences (teleological mode). Jamie's parents are more able to see situations from Jamie's perspective and use effective communication and consequences to scaffold her independent problem solving. Put differently, Jamie's identity and personality development are being supported and she is learning to make choices in her own best interest.

Jamie's predisposition toward intense emotionality and impulsivity may become difficult for her to manage again in the future if she encounters difficulty without adequate support from her attachment figures or other social relationships. In such times, Jamie may find it useful to engage in treatment again to gain support to manage these new developmental demands.

### 2.8 Clinical Practices and Summary

In summary, we have presented a case illustration using MBT‐A principles with special consideration of the developmental aspects of adolescents which should be taken into account by clinicians. In addition, we have incorporated important innovations of MBT‐A which increase its utility. First, in terms of nosological reform, we have described the benefits of conceptualizing personality challenges in terms of self and interpersonal functioning, rather than categorical BPD symptoms. This difference in nosology communicates clearly to the teen and family where the root of the teen's difficulties lies and how they can improve them. By keeping emphasis on the serve‐and‐return between an adolescent and their caregiver, the desired outcome of MBT‐A can be achieved: enhanced mentalizing capacity in the adolescent in their natural home environment. Second, we emphasized the importance of mentalizing as a transdiagnostic construct which is useful to be considered across all psychopathology. The mentalizing stance opens the door for learning across all psychotherapy modalities and for all mental health problems. For instance, the self and interpersonal functioning of an adolescent struggling with anxiety, depression, or impulsivity is highly significant to treatment. By utilizing mentalization‐based components, such as the mentalizing stance, a therapist doing standard cognitive‐behavior therapy will be able to make better use of the therapeutic relationship. Third, we emphasized the importance of enhancing mentalizing outside of the therapy room. While the therapist acts as an important facilitator of the adolescent's mentalizing, the ultimate goal is for the adolescent to be able to make use of their relationships in their home environment with peers, parents, teachers, and others. A mentalization‐based therapist may lay the foundational work to open the epistemic highway which allows the adolescent to be open to learning from them. However, the desired outcome of MBT‐A is for an adolescent to benefit from the naturally useful effects of their relationships with others. We hope clinicians will make use of this case illustration to bring mentalization‐based theory and components into their own work with adolescents.

### 2.9 Limitations and Suggestions for Future Study

This naturalistic case study contains several limitations. As a naturalistic case study, this patient does not contain a control condition and therefore has potential for bias. In addition, the use of tools for baseline, process and outcome assessment is encouraged in the MBT‐A framework. For additional information on this, see for instance Rossouw et al. (2021). Additionally, as the therapist in this case study is a graduate student, there may be impact of training‐related variables such as competence and adherence. However, as she was supervised by a certified MBT‐A clinical supervisor, we argue that the present case study may be useful to other clinicians. In the future, more research is needed to examine the impact of each MBT‐A innovation on its own (MBT‐A dismantling study). It may also be useful to consider a stepped‐care approach in MBT‐A to examine which populations and disorders benefit most from various MBT‐A models. Overall, we believe our case study contains several strengths including a useful clinical illustration of recent advancements in MBT‐A.

## Ethics Statement

This study was conducted in accordance with recognized ethical principles for case study research. Informed consent was obtained from the participant and their parents for inclusion in the present study. To ensure confidentiality, pseudonyms were used in all records and reports, and identifying details were removed or altered. Data were stored securely and used solely for research purposes.

## Conflicts of Interest

The authors declare no conflicts of interest.

## Data Availability

Data sharing not applicable to this article as no datasets were generated or analyzed during the current study.
